# Recent advances in polygenic scores: translation, equitability, methods and FAIR tools

**DOI:** 10.1186/s13073-024-01304-9

**Published:** 2024-02-19

**Authors:** Ruidong Xiang, Martin Kelemen, Yu Xu, Laura W. Harris, Helen Parkinson, Michael Inouye, Samuel A. Lambert

**Affiliations:** 1 Cambridge Baker Systems Genomics Initiative, Baker Heart and Diabetes Institute, Melbourne, VIC Australia; 2 Cambridge Baker Systems Genomics Initiative, Department of Public Health and Primary Care, University of Cambridge, Cambridge, UK; 3 British Heart Foundation Cardiovascular Epidemiology Unit, Department of Public Health and Primary Care, University of Cambridge, Cambridge, UK; 4 Victor Phillip Dahdaleh Heart and Lung Research Institute, University of Cambridge, Cambridge, UK; 5 Health Data Research UK Cambridge, Wellcome Genome Campus and University of Cambridge, Cambridge, UK; 6 European Molecular Biology Laboratory, European Bioinformatics Institute, Wellcome Genome Campus, Hinxton, Cambridge, UK; 7 British Heart Foundation Centre of Research Excellence, University of Cambridge, Cambridge, UK

**Keywords:** Polygenic score (PGS), Clinical utility, FAIR (Findable, Accessible, Interoperable, And Reusable), Genome-wide association studies (GWAS), Open-access, Transferability, Responsible use

## Abstract

Polygenic scores (PGS) can be used for risk stratification by quantifying individuals’ genetic predisposition to disease, and many potentially clinically useful applications have been proposed. Here, we review the latest potential benefits of PGS in the clinic and challenges to implementation. PGS could augment risk stratification through combined use with traditional risk factors (demographics, disease-specific risk factors, family history, etc.), to support diagnostic pathways, to predict groups with therapeutic benefits, and to increase the efficiency of clinical trials. However, there exist challenges to maximizing the clinical utility of PGS, including FAIR (Findable, Accessible, Interoperable, and Reusable) use and standardized sharing of the genomic data needed to develop and recalculate PGS, the equitable performance of PGS across populations and ancestries, the generation of robust and reproducible PGS calculations, and the responsible communication and interpretation of results. We outline how these challenges may be overcome analytically and with more diverse data as well as highlight sustained community efforts to achieve equitable, impactful, and responsible use of PGS in healthcare.

## Introduction

Genome-wide association studies (GWAS) have linked genetic loci across the genome with many hundreds of diseases and quantitative traits [1, 2], and found that many of these complex traits have a polygenic architecture, where phenotypic variance is accounted for by many genetic variants of small effect. GWAS information, either individual-level or summary statistics, can be leveraged to estimate an individual’s genetic predisposition for a given phenotype [3–9]. This genetic predisposition is typically represented as a score and is referred to as a polygenic score (PGS), polygenic risk score (PRS) or genetic/genomic risk score (GRS). PGS are based on cost-effective technology (e.g. genome-wide genotyping array or sequencing) which, since it is measuring the germline genome, only needs to be performed once in an individual’s lifetime. Further, PGS for hundreds of diseases and/or clinically relevant traits can be calculated from one genome-wide array or sequence.

For many clinical use cases, PGS are being evaluated around the world to determine what clinical utility they may have. For example, Genomics PLC and GP practices in the North of England are piloting PGS as part of an integrated risk tool for cardiovascular risk assessment [10]. The PGS-augmented CanRisk tool [11] for breast and ovarian cancer is being evaluated as part of the PERSPECTIVE I&I study [12], and additional trials of PGS-augmented integrated risk tools (IRTs) for breast cancer are in progress, including WISDOM [13] and MyPEBS [14]. The GPPAD, PLEDGE and CASCADE trials are evaluating PGS for use in autoantibody screening of type 1 diabetes [15]. In the USA, multiple studies are ongoing for how returning genetically-informed risk information using PGS for multiple diseases impacts outcomes in individuals of diverse ancestries, such as the Genomic Medicine at Veterans Affairs (GenoVA) [16] and electronic MEdical Records and GEnomics (eMERGE) studies [17]. Large-scale biobanks and infrastructures are also accelerating the speed of development and translation for PGS (e.g. UK Biobank), and the next generation of genomic cohorts are well-placed to widen both the scale, demographic diversity and power of PGS (e.g. All of Us Program [18] and Our Future Health [19]).

The translation and clinical implementation of new tools is challenging, and this has been particularly the case for PGS. The technologies on which PGS depend, genotyping arrays and sequencing, are largely yet to make their way into routine healthcare. Genotyping arrays have seen slow clinical adoption while whole genome sequencing has had several major applications for the genomic surveillance of microbial pathogens [20], cancer genomics [21] and diagnosis of rare developmental disorders [22]. The breadth of potential clinical applications for PGS combined with other risk factors is extensive, yet there are common challenges. Here, we review the potential benefits and challenges facing the implementation of polygenic scores in clinical practice. In doing so, we highlight a series of important findings which may guide future clinical research in evaluating the utility of PGS.

## Potential benefits of polygenic scores

### Disease risk prediction alongside other risk factors

PGS have the potential for clinical utility as they measure aspects of disease risk that are independent of or precede traditional risk factors [6] recent studies have expanded the evidence in this area. Genetic predisposition to disease can be partially captured by family history; however, family history is a composite variable that captures both shared environment and genetic similarity that is often incomplete and poorly captured [23]. As such, PGS has been shown to add information beyond family history in phenotype prediction for a child based on the average of their parents (mid-parent) for traits like height [24, 25] and risk of common diseases [24, 26]. Family history may also correlate with the presence of familial forms of disease caused by rare pathogenic variants, and most genetic tests implemented in current clinical practice assess a variant’s occurrence in familial and sporadic disease cases. However, there is significant heritability outside of rare variants which is quantified by the common genetic variants comprising PGS, which can predict sporadic cases of polygenic disease [27]. As such, PGS has been shown to add additional risk stratification in individuals with high genetic risk for diseases including type 1 diabetes [28] and BRCA1/2 carriers [29, 30].

Many diseases have multiple biological, environmental or lifestyle risk predictors that are combined into risk prediction models. These conventional risk predictors frequently include age, sex, body mass index (BMI), smoking behaviour, family disease history and established clinical assays [31]. However, many models have disease-specific predictors. Various studies have found that, when treated the same as other risk factors, PGS contributes independent information that improves the accuracy of these risk prediction models [6], and studies continue to show that PGS modestly improve risk prediction when combined into an IRT for diseases of major public health burden, including coronary heart disease [32, 33], stroke [34, 35], type 2 diabetes [36, 37], and breast cancer [38]. Improvements in risk prediction have frequently been shown in terms of classification accuracy (e.g. ‘high’, ‘intermediate’ or ‘low’ risk groups which correspond to different clinical recommendations), leading to the conclusion that PGS only modestly improves risk stratification. However, it is important to highlight that *prima facie* small changes in overall classification accuracy can translate into meaningful benefits at scale. For example, Sun et al. [32] showed that adding PGS of coronary artery disease (CAD) and ischaemic stroke [39, 40] to conventional risk factors resulted in increases in classification accuracy of 1–2% (ΔC-index); however, the addition of PGS improved continuous net reclassification for 10% of incident cardiovascular disease cases and 12% of non-cases, yielding an additional 72 prevented cases per 100,000 adults, per 10 years.

**Fig. 1:**
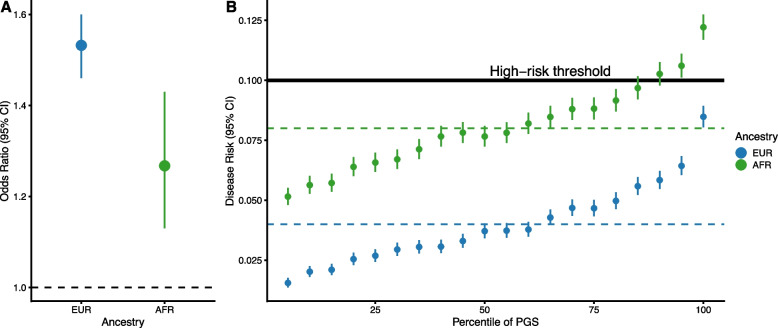
Baseline risk can substantially change the utility of a polygenic score. **A** Effect size (odds ratio) of PGS for an example disease in the populations of European (EUR) and African (AFR) ancestry. **B** Prevalence of disease risk across PGS percentiles. The bolded line indicates a high-risk threshold that impacts treatment decisions (here 10%, similar to most clinical guidelines [53]). Dashed lines indicate the average disease risk in each ancestry group. Data presented are simulated [54] to match observed effect sizes for PGS for CAD [52] and assuming that the African population has a two-fold higher disease risk than the European ancestry population (here with a baseline risk of 4%) similar to the observed difference in cardiovascular disease incidence between ethnicities [55]

The Sun et al. study carefully evaluated PGS in the context of baseline risks which mirrored demographics in primary care (i.e., correcting for the healthy participant bias in UK Biobank); however, despite being integral to the clinical utility of risk prediction, baseline risk is frequently forgotten in PGS studies. Baseline risk can be critical for apparently modest predictors like PGS, especially in groups with otherwise high baseline risk (Table 1, Fig. 1).

**Table 1:** A focus on difference in PGS classification accuracies between groups can mask potential utility when baseline risks differ

Many critiques of PGS focus on their modest effect size (e.g., odds or hazard ratio) and the risk stratification between the top and bottom quantiles of genetic predisposition, which is related to the proportion of variance explained (*r* ^2^) and classification accuracy (AUROC or C-index) [41, 42]. As noted, the risk stratification capacity of PGS decreases proportional to the genetic distance from the training population, leading to attenuated but non-null effect sizes in non-European ancestry groups [43] (specific analyses of PGS for coronary artery disease or CAD [44], breast [45] and prostate cancer [46]). Given that these effect sizes (and thus classification accuracies) are non-null indicates that they may still be useful for stratification. This may be particularly true when the baseline risk is higher in the groups with lower effect sizes, and it is the case that many non-European ancestry groups have a significantly higher incidence for some common diseases [47, 48]. In cases where demographic groups have different average/baseline risks, it can be difficult to infer clinical utility of a PGS when looking at PGS alone [49] (Fig. 1).
Existing European-biased PGS integrated into clinical risk tools have been shown to improve the reclassification of cases in non-European ancestries in multiple studies of cardiometabolic diseases [33, 37, 50–52]. In these circumstances, higher baseline risk can compensate (partially or completely) for attenuated PGS performance with respect to metrics of disease prevention in each group (e.g. number of events prevented, number needed to treat/screen to prevent one event, etc.).
While analysis of risk stratification and utility is more interpretable using absolute rather than relative risk differences, it of course does not address the underlying representational bias in the data. Global efforts to collect genomic data in more diverse cohorts should certainly continue and form the foundation for greater equity in the future.

Age is the strongest risk factor for most common diseases and contributes to the baseline risk profile along with the accumulation of other risk factors. As such, analyses show that PGS is a stronger predictor of disease incidence earlier in the life course [33, 35, 56, 57], which motivates measuring genetics earlier in life for targeted prevention and screening before the accumulation of risk factors and environmental exposures. While age affects genetic relative risk for many common diseases as captured by PGS [57], a recent study of prostate cancer illustrates the utility of PGS for absolute risk-stratification despite age-related attenuation of relative risk [58]. Taken together, there is now a strong evidence base across many diseases that PGS captures disease risk information that is independent of other risk factors and improves integrated risk calculators. 

### Assessing the clinical utility of polygenic scores

The utility of a PGS ultimately depends on its predictive ability and the clinical scenario in which it is applied [59]. Here we highlight examples of clinical scenarios where a PGS has been proposed to have the potential for utility.

#### Risk stratification

For many diseases, PGS may be useful for risk stratification as they tend to be more informative earlier in life, and those of different genetic predispositions will be predicted to become high risk at different ages. As is the case with other risk factors, disease prevalence may affect the performance of a PGS — for example, for a disease with a prevalence of 1% (e.g. schizophrenia), the top 10% of a current PGS would only identify 3% of patients [60, 61]. However, PGS can still be useful for risk stratification in high-risk groups of low-prevalence diseases (e.g.T1D [28, 62]), or used in combination with other risk factors to define a higher-than-average risk population in which to screen. Thus, PGS may be useful for changing the age and/or frequency at which people are screened for cardiovascular risk factors, common cancers (e.g. breast, prostate, colorectal), and other conditions (e.g. dementias). The benefits of using PGS to optimize cancer screening have been shown to be cost-effective [63, 64], but more evidence is needed and multiple trials assessing outcomes and feasibility are ongoing (WISDOM [13], MY-PEBS [14], BARCODE [65]). Similar analyses of PGS in cardiometabolic diseases also indicate clinical benefits and cost-effectiveness [66]. While cost-effectiveness studies of PGS are still emerging, few assess multiple disease use cases and thereby do not account for the fact that a single array/sequence could marginally improve risk stratification for multiple diseases simultaneously.

Risk stratification based on IRTs that include PGS may also be used to guide treatment decisions, including pharmacological interventions. Multiple studies have shown the potential benefit of adding PGS to cardiovascular disease calculators and that combined models identify significant numbers of additional future cases surpassing risk thresholds to receive statins, the most common risk-reducing medication for atherosclerotic disease [32, 67]. Indeed, benefit estimation may need to take into account the potential for effect modification of polygenic risk on treatment effectiveness as multiple studies have shown that individuals at high polygenic risk of CAD may benefit disproportionately from the use of statins or PCSK9 inhibitors in terms of relative and absolute risk reductions (see below) [66, 68–70].

Behaviour change in humans is frequently difficult to achieve and the impact of phenotypic or genetic risk score information is no exception [71]. While more follow-up will ultimately determine whether changes in behaviour are persistent and corresponding disease events reduced, recent large-scale studies suggest IRTs including PGS may motivate positive changes to modifiable risk factors. Results from the GeneRISK in Finland study showed that after 1.5 years of interacting with an online CVD risk communication tool integrating PGS, 42.6% of 7342 participants at high risk had made positive health behavioural changes, including weight loss, quitting smoking or becoming a member of online health coaching services [72]. This is to be contrasted with other studies such as INFORM which have assessed, in a randomized trial, whether provision of genetic or phenotypic risk scores cause positive behaviour changes [73]. Exemplifying the difficulty of affecting human behavioural change, the INFORM trial found no significant effects for either genetic or phenotypic scores. Importantly, the studies did not find anxiety and depression in response to PGS information to be common.

CanRisk is a web tool for the Breast and Ovarian Analysis of Disease Incidence and Carrier Estimation Algorithm (BOADICEA), which combines PGS with conventional risk factors like age, family history, mammographic density and known pathogenic variants [74–76]. CanRisk is CE marked and is an early implementation of PGS for clinical use. When only using questionnaire-based risk factors and mammographic density, BOADICEA identifies 9.2% of women with moderate to high-risk [74]. The 313-SNP PGS [74] for breast cancer alone identifies 10% and when the PGS is added to BOADICEA, the integrated model identifies 13% of women in moderate to high-risk [74, 76]. The CanRisk model is amenable to updates using other PGS [77] and estimates from CanRisk can be used to guide screening and choices of risk-reducing interventions, including surgical procedures (e.g. UK’s NICE guidelines [78]).

#### Diagnosis

In some diseases, patients with severe and early onset disease undergo genome analysis to identify a putative genetic cause. Currently, gene panel testing is the most common type of testing, with exome and whole-genome sequencing being increasingly applied to challenging cases to improve diagnostic yield [79, 80]. For example, of 60 patients from a preventative genomics clinic (both self-referred and referred by cardiologists) [81], two had a monogenic variant for familial hypercholesterolemia (i.e. classified as high monogenic risk), but 19 had a PGS in the top quintile. Lu et al. [82] showed that PGS can discriminate high familial monogenic risks for breast cancer, bowel cancer, heart disease, type 2 diabetes and Alzheimer's disease [82]. Their study demonstrated that PGS may be able to prioritize patients for subsequent diagnostic sequencing, which may increase cost-effectiveness. While rare pathogenic variants are clearly disease-causing, the majority of common disease cases will not have one of these variants, and a polygenic aetiology (e.g. presence of a ‘high’ PGS) will be more likely [83]. Of course, scores integrating the full spectrum of allele frequencies will likely be optimal [84] and the development of methodologies to construct PGS that include rare variants is an active area of research [85–87]. There are also clinical scenarios where PGS might be useful for differentiating between possible diagnoses, e.g. discriminating type 1 diabetes from type 2 diabetes [88] or MODY [62]. For ankylosing spondylitis (AS) and individuals who present with back pain, a PGS had the highest classification accuracy, compared to MRI scans or HLA-B risk allele status, to distinguish AS cases and non-AS ([89]). PGS in autoimmune diseases frequently exhibit higher classification accuracies than other diseases (e.g. AUROC > 0.9 [28]), likely due to high heritability and the combination of large effect-size HLA variants, illustrating their potential utility for improving screening pathways.

#### Use in clinical trials and for understanding treatment benefits

As outlined by Fahed et al. [90], PGS also have potential uses for assessing the benefits of pharmacological therapies. Clinical trials can be large in scale and expensive to run in order to accumulate the numbers of outcomes to measure an effect; thus, to achieve this, trials often enrol individuals at high risk of the outcome. Fahed et al. showed how using a PGS might reduce trial sample size by focusing on individuals at high polygenic risk to increase the outcome rate. A PGS-guided trial strategy might be especially useful for preventative interventions in high-risk individuals before disease onset (e.g. before cognitive impairment in dementias or Alzheimer’s disease [91, 92]) or in those individuals who are susceptible to T1D [28]. Notably, PGS-based enrichment of trials may result in more efficient trials but they would require participants to be genotyped prior to enrolment. Emerging population-scale platforms (such as Our Future Health in the UK) may enable such PGS-guided trials.

Retrospective genetic analyses of clinical trials for multiple cardiovascular disease treatments have also shown that treatment benefit may be greatest for those at high polygenic risk, including the FOURIER trial [69], Odyssey Outcomes trial [70] and statin therapy [68, 93, 94]. This is consistent with observational data, where PGS but not clinical risk factors were shown to stratified populations most likely to benefit from treatment (59% vs. 33% relative risk reduction for incident myocardial infarction in the highest and lowest genetic risk groups respectively) [68]. Targeting treatments to those most likely to benefit would be advantageous [95], particularly for treatments that are costly. While high profile studies have been performed in cardiovascular disease, PGS have been shown to have potential to predict treatment responses to other conditions, including migraine [96], type 2 diabetes [97] and psychiatric disorders [98] like schizophrenia [99], and depression [100]. Overall, PGS could prove useful for designing more efficient trials as well as for identifying those most likely to benefit from specific treatments.

## Analytic challenges for translation of polygenic scores

PGS are moving toward clinical implementation in many scenarios. As such multiple consortia of researchers and clinicians have put forward guidance on the use and interpretation of PGS, these include a statement from the Polygenic Risk Score Task Force of the International Common Disease Alliance (ICDA) [9], and the American College of Medical Genetics and Genomics (ACMG) [101]. In this section, we highlight key analytic challenges, possible solutions, and linkages across translational efforts.

**Fig. 2:**
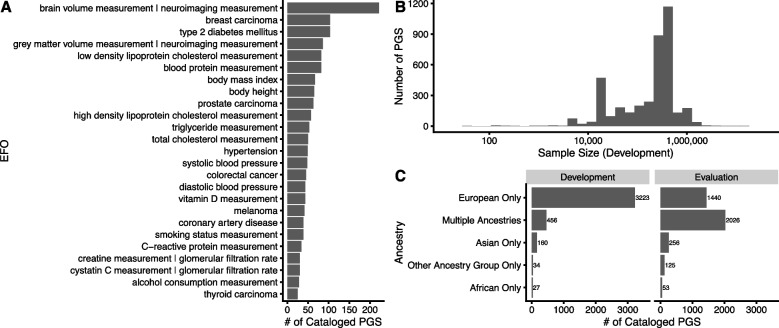
Summary of publicly available PGS. **A** Top 25 traits/diseases which have the greatest number of PGS in the PGS Catalog. **B** Distribution of total sample size (sum) used to develop each PGS (either as a GWAS or in score development). **C** Ancestry composition of sample sets used for PGS development and evaluation for each PGS. All evaluation samples were aggregated to define the final label. Data was extracted on December 7, 2023, with a total of 3900 PGS with catalogued IDs for 619 traits from 507 publications

Developing, calculating, and applying PGS is a data-intensive endeavour, and should strive to be Findable, Accessible, Interoperable and Reusable (FAIR, [102]) in order to maximize PGS reproducibility and utility as research and potentially clinical tools. PGS are typically constructed using coefficients from GWAS, and as such it is critical that the GWAS summary statistics are openly shared and reusable by other researchers. Sharing data via a recognized repository, such as the GWAS Catalog [2], where data is stably accessioned and made available in a standard format facilitates the linking of PGS to source data. High-quality study and variant-level metadata in GWAS summary statistics (e.g. imputation INFO scores, allele frequencies, and per-variant sample sizes) are required for accurate PGS development and input to many methods [103]. As many fields are underreported in shared GWAS summary statistics (e.g. allele frequency), submitters are encouraged to format and openly share these data according to recently established community standards [104]. The information necessary to reproduce PGS (e.g. the variants and weights) should also be shared, thereby enabling independent evaluations in new cohorts and comparison to newly developed PGS. To facilitate the open sharing of PGS, Lambert et al. [105] developed PGS Catalog (https://www.pgscatalog.org/). Currently, the PGS Catalog has catalogued ~ 4000 scores predicting ~ 600 different complex traits and/or diseases from ~ 500 publications (Fig. 2). Alongside the PGS Catalog, the Polygenic Risk Score Reporting Standards (PRS-RS) [106] have outlined key performance metrics and considerations for PGS analyses as reporting has been highly heterogenous. Both GWAS and PGS should be shared with clear and unambiguous license terms (ideally CC0 or, if necessary, CC-BY-NC) to ensure reusability for the widest range of research and clinical applications.

Although biased by the availability of PGS that have been added to the PGS Catalog (see inclusion criteria https://www.pgscatalog.org/about/inclusioncriteria), European ancestries still comprise the plurality of PGS training and prediction samples, followed training samples combining data from multiple ancestry groups and then a much smaller number of Asian ancestry studies (Fig. 2C), highlighting that ancestral diversity is a problem for PGS, consistent with other systematic reports [107]. This lack of ethnic, ancestral and demographic diversity is observed in many epidemiological and clinical studies, including the vast majority of the GWAS which underpin the training of PGS.

### Improving the transferability of polygenic scores across ancestries

A key challenge for the utility of PGS is to ensure they make equitable predictions for all groups; however, many PGS have weaker predictive performance between populations defined by their genetic ancestry [108] and within some sub-groups of a single ancestry group [109]. This issue, which is common to other biomarkers and risk models, is often called the transferability (or portability) gap and, in this case, refers to the relative predictive ability of a PGS in samples that are external to the PGS development populations. It should be noted that some attenuation of predictive performance (e.g., effect size, accuracy, R^2^) is expected and can be based on differences between the training cohort and that being evaluated (e.g., demographic differences, social determinants of health, case ascertainment/phenotyping), which is why external validation is a critical step in any risk model evaluation [106, 110]. It is also well-documented that the attenuation in PGS predictive ability is proportional to the genetic distance from the training population [111, 112]. Over 95% of recent GWAS study participants have been of European ancestry [107, 113]. Several recent reviews [5, 114–116] have also acknowledged the transferability issue of PGS.

Multiple studies have shown that more diverse and multi-ancestry GWAS can improve the predictive power and transferability of PGS, likely because the effect sizes of the true causal variants are shared across ancestry groups [117]. For example, a recent study of blood lipid levels showed that PGS constructed using multi-ancestry GWAS outperforms those constructed using single-ancestry matched data [118]. A larger analysis of 14 disease endpoints results from the Global Biobank Meta-analysis Initiative (GBMI) also concluded that using multi-ancestry GWAS improved the accuracy of PGS for all ancestries, although a significant amount of heterogeneity in accuracy exists across ancestries [119], and many other PGS based on multi-ancestry GWAS can be validated in diverse populations [46, 120, 121]. However, multiple studies constructing and evaluating PGS in African populations have come to the opposite conclusion that ancestry-matched PGS is most optimal for the prediction [25, 115, 122, 123]. One reason for this could be that not all traits are perfectly genetically correlated across ancestry groups [124], with notable examples for psychiatric disorders [125, 126].

More transferrable PGS can also be developed by using improved statistical methods (see [4] for a recent comprehensive review of PGS development methodologies). The major advancements used to close the transferability gap are primarily based on ensembling and leveraging multi-ancestry and multi-trait data and incorporating functional information to identify more likely causal variants. Ensembling-based methods are based on the idea that incorporating multiple sets of GWAS data from either multiple ancestries or multiple diseases/risk factors can create a better set of variants and weights for PGS calculation. One such approach is PRS-CSx [127], an extension of the population PRS-CS continuous shrinkage (CS) models that can be shared across ancestries. Another example of ensembling is CT-SLEB [128] which integrates clumping and thresholding, empirical Bayes and super learning to process multi-ancestry GWAS data into a single PGS. More complex methods that calculate and normalize PGS based on variants in local ancestry blocks are also being developed [129]; however, the complexity of software implementation will be a challenge as they also require sharing of reference panels for chromosome painting.

As causal variants are similar across ancestries [117, 130], it is possible that PGS based on these causal variants may yield more similar prediction performances. Causal variants are expected to have relevant biological functions, thus such information can be used as biological priors to better select variants and then train better weights [131]. Multiple methods using biological information/annotation have been shown to improve the transferability across ancestries, including LDpred-funct [132], PolyPred + [133] and BayesRC [134]. Simpler methods exist to use relevant annotations for variant selection and use GWAS effect sizes [135]. Since integrating GWAS summaries from multi-ancestries and leveraging SNP annotation both improve the transferability of PGS, combined approaches such as X-Wing [136] and PolyPred + [133] may significantly improve PGS accuracy in non-European populations [136].

The differences in PGS accuracy that can be observed between genetically defined populations can be related to differences in effect sizes, LD patterns and allele frequency patterns, but they can also be due to correlations with other factors. For example, the accuracy of PGS within African populations was found to be low but highly variable between different ethnic groups of Sub-Saharan Africa [137], which may be due to the correlation between ancestry groups and social determinants of healthcare, selection and the differential impacts of genetics in different environments [5, 109, 138].

### Reliable and reproducible PGS: assays and computational pipelines required for implementation and interpretation

PGS development results in a set of variants and weights that can be used to estimate genetic predisposition; however, other steps are necessary to measure PGS in individuals and return an interpretable test result [59]. Typically PGS have been developed in cohorts of genotyped individuals using a limited set of directly measured variants on a genotyping array, which has been imputed to higher genome coverage using reference panels [139]. Recent studies have shown that the choice of imputation panel and strategy can affect PGS accuracy [140], and the choice of genotyping array can be particularly important for underrepresented populations [141]. Ideally, the clinical use of PGS should combine common and rare variants [139], even if the improvements to risk-stratification at the population level may be limited [142]. However, as rare variants are difficult to impute accurately [143], they are usually excluded from PGS development and/or calculation [139]. A potential solution is to use whole genome sequencing; however, the cost of whole genome sequencing still inhibits large-scale implementation. An alternative is low-coverage sequencing (< 1 × coverage) coupled to genotype imputation, which is more scalable and improves the accuracy of PGS calculation as compared to genotyping arrays [144–146].

Another challenge in PGS calculation is that scores are often on different scales (different mean and variance), and different genetic ancestry groups can have shifted PGS distributions that do not reflect differences in the disease prevalence [147]. Thus the main way to convert a PGS into an interpretable individual measure is to represent it as a relative risk of where an individual sits in a population distribution. In a cohort of genetically similar individuals, one can simply normalize the PGS for the mean and standard deviation of the population of interest or use percentiles; however, this becomes challenging for diverse ancestries and/or admixed individuals. One way to calculate an individual’s PGS is to use a population reference panel (e.g. 1000 Genomes Project) and report an individual's relative PGS with respect to the most similar population in the panel. Recent methods have been proposed that do not rely on reference population labels as they use the associations of PCA loadings to PGS values to decorrelate PGS distributions from genetic ancestry. Initially, these methods only corrected for different mean distributions in PGS distributions [148], which has been implemented for PGS reporting in the GenoVA Study [16]. However, differences in the variance of PGS distributions between populations can also be corrected by regressing the variance of the new PGS distribution with the PCs [149] — this was used to report PGS information within the eMERGE study’s genome-informed risk assessment (GIRA) report [17]. All three methods of normalizing PGS (using empirical distributions, or using PCA loadings to centre the mean and equalize variance) result in a relative risk to a population average and can be reported as is for interpretation (e.g. polygenic risk reports) or as a predictor in risk tools. Overall considerations for how to report a PGS depend on choices of genotyping assay, imputation, and how a PGS is calculated/adjusted, and these all have implications for how it is regulated and reported [59, 106].

### Ensuring the responsible use, communication and interpretation of PGS

Polygenic risk ultimately has to be communicated to many different stakeholders, including patients (and/or consumers of commercial genetic testing) and clinicians if they are to be used in the clinic. Understanding of PGS among these groups may be low, so effective PGS reports and communication [150] are critical [114, 151] — some examples of reports being used to communicate PGS results already exist [17, 152]. Notably, it is important that PGS reports/results do not convey genetic determinism (that genetics predictions are *fait accompli*) or exceptionalism (that genetic predisposition is more important than other risk factors). However, information about how the estimate was developed is just as important as the risk estimate itself, e.g. the population(s) used to develop and train the score is critical for interpreting whether the risk estimate is applicable to the individual at hand [101]. Adherence to reporting standards and key metadata requirements describing how the PGS was developed and evaluated can achieve this goal [106, 153], as different studies often report PGS metrics with different statistics and covariate adjustments that make comparisons difficult. During the reporting of PGS/IRT development, it is important to describe participant inclusion, as the labels we use to describe populations can be imprecise or comprise outdated language that can cause harm and misinterpretation (see NASEM review [154]). Consistent with what many have advised, the NASEM report recommended that we should not use race as a proxy for human genetic variation nor as part of PGS, and one should carefully consider any labels applied when grouping individuals. This is especially important as most PGS studies compare effect sizes and accuracies across groups, usually labelled according to their continental ancestries which individuals might not identify with. The use of continental ancestry descriptors also causes problems as researchers do not always consider the genetic diversity within these populations, and examples of fine-scale genetic structure impacting PGS calculation exist [155–157]). Methods used to calculate PGS as a relative risk often also rely on matching individuals to a reference population/label; however, promising improvements to normalize PGS using continuous measures of genetic ancestry derived from reference panels are outlined above [148, 149], and can avoid the use of labels that can differ from how a person identifies [158].

Consistent with the views of the vast majority of the PGS research community, the ACMG’s statement advocates against using PGS as a standalone test, as a negative result is not conclusive, and a positive result does not always mean the carrier is at high immediate risk. As we have highlighted above, except in some diseases such as autoimmunity (e.g., [159]) or Alzheimer’s disease (e.g., [160]), PGS are frequently modest standalone predictors of disease risk — their main advantage is that they capture risk information that is not being measured already using genetic testing or traditional risk factor models. The ACMG also outline that PGS should be combined with genetic testing for rarer pathogenic variants or those causing monogenic disease, as well as combining PGS with other clinical measurements to understand a patient's current health status and the examples of PGS utility we summarized in this review mainly implement PGS alongside currently implemented risk estimation and rarely in isolation. Both the ICDA and ACMG statements also outline a shared goal of making sure that PGS are used equitably and that methodological development and data collection should be advanced to ensure PGS work optimally in all individuals regardless of their genetic ancestry. This also includes making sure that PGS is not used in any situations that might cause harm or otherwise be unethical. A significant gap in the literature exists to define what is best practice when an individual engages a healthcare practitioner with PGS results which they have obtained from a third-party provider (commercial or otherwise). Anecdotal reports indicate this is no longer a rare event. While not the focus of this review, parallel statements have been released calling for an end to the use of PGS for embryo selection [161–163] or for unscientific claims about racial/ethnic group differences.

## Conclusions and future directions

The evidence for the clinical utility of PGS is continuously developing, but PGS are already used in some risk tools implemented in clinical practice, and select preventative genomics clinics. In the near-term, it is likely that the continued deployment of PGS in clinics will rely on extending conventional risk models into integrated risk tools enhanced by PGS (e.g. CanRisk [11]). Despite their manifold potential benefits, PGS have inherent risks and limitations, similar to other risk factors, such as variable portability across genetic ancestry groups. While improvements to PGS development methods can partially overcome these limitations, the only genuine solution is to increase the representation of diverse samples in the GWAS [122]. The open sharing of this genomic data and the developed PGS should be openly shared according to FAIR principles [102] and established reporting guidelines [106] to maximize equitable translation of these results. There is also additional work to be done to develop best practices for calculating individuals' PGS, both in genotyping assay and/or imputation choices and how to calculate and report a person’s risk. As with many tools already utilized for disease risk prediction (e.g. QRISK [10]), there is an absence or paucity of randomized trial evidence as to their clinical benefit and there are various reasons for this, e.g. the vast number of PGS, clinical use cases, number of patients needed and corresponding scarce resources [164]. Alongside efforts to conduct pragmatic trials of PGS, large-scale validation together with rigorous clinical and population health modelling should continue. Health economic modelling and feasibility studies will also inform decisions of whether PGS implementation should proceed in any particular use case. Following from these requirements is the need to communicate how the full PGS development, evaluation and calculation has been performed so that ensures it is understandable to physicians and patients. Importantly, significant community efforts should be invested to ensure the responsible use of PGS to counter genetic determinism and exceptionalism.

While analytic solutions (including methods development and modelling of baseline risk) are being developed, the lack of diverse genomic data continues to be an important limitation. While it will take substantial time to recruit participants from historically underrepresented groups and to generate genomic data, the most effective strategy at the moment is to leverage multi-biobank resources, e.g. the Global Biobank Meta-analysis Initiative [119, 165]. Many wealthy countries like the USA and UK are recruiting and delivering the next phase of larger and more diverse biobanks (e.g. the All of US Program [18] and Our Future Health [19]). However, continued efforts should ensure that the benefits of PGS are not only available to those in wealthy countries and capacity building and ethical partnerships for data collection and analysis in underrepresented groups, particularly low-middle income countries, should be promoted [166–170]. Statistical methods, tools and resources also should be improved to facilitate analysis of genetic ancestry on a continuum, particularly so that admixed individuals are not excluded from studies [154, 171]. The standardization of GWAS/PGS results reporting, responsible use and communication will require a concerted effort from academic, industry and government bodies. Overall, through community efforts towards common goals, it is clear that continued progress in PGS is being made and that it could benefit human health. There is now a substantive need for further translational studies, including pragmatic trials, to provide empirical evidence as to PGS utility in specific clinical scenarios.

## Data Availability

The summary data used from the Polygenic Score Catalog [105] is publicly available at https://www.pgscatalog.org/.

## Abbreviations

ACMG: American College of Medical Genetics and Genomics
AS: Ankylosing spondylitis
AUROC: Area under the ROC (receiver operating characteristic) curve
BMI: Body mass index
BOADICEA: Breast and Ovarian Analysis of Disease Incidence and Carrier Estimation Algorithm
CAD: Coronary artery disease
eMERGE: Electronic MEdical Records and GEnomics study
FAIR: Findable, Accessible, Interoperable, and Reusable
GBMI: Global Biobank Meta-analysis Initiative
GenoVA: Genomic Medicine at Veterans Affairs study
GIRA: Genome-informed risk assessment
GRS: Genetic/genomic risk score
GWAS: Genome-wide association
ICDA: International Common Disease Alliance
IRT: Integrated risk tools
NASEM: National Academies of Sciences, Engineering, and Medicine
PGS: Polygenic score
PRS: Polygenic risk score
T1D: Type 1 diabetes
